# It’s how we practice that matters: professional identity formation and legitimate peripheral participation in medical students: a qualitative study

**DOI:** 10.1186/s12909-022-03107-1

**Published:** 2022-02-09

**Authors:** Paul Orsmond, Helen McMillan, Remigio Zvauya

**Affiliations:** 1 College of Medical and Dental Sciences, University of Birmingham, Birmingham, B15 2 TT UK; 2 Staffordshire Centre of Learning and Pedagogic Practice, Institute of Education Brindley Building, Staffordshire University, ST4 2DF Stoke-on-Trent, England

**Keywords:** Graduate entry, Early clinical experience, Communities of practice, Professional identity, Legitimate peripheral participation

## Abstract

**Background:**

The process of Legitimate Peripheral Participation (LPP) within a community of practice framework (CoP) was used to explore graduate entry medical students’ professional identity formation (PIF) during their first year of study. A conceptual model has been developed that can be used by medical educators to better understand PIF and to aid the explicit incorporation of PIF activity within the undergraduate curriculum.

**Methods:**

Ten students from one UK medical school participated in the longitudinal study and were interviewed at three points during the first year. Semi-structured group interviews were used to explore students’ experience of the clinical environment and the nature of their interactions with both clinicians and patients in a community-based medicine practice. The interviews were audio recorded and transcribed. Thematic analysis was used to identify overarching themes which are represented as facets in the model of PIF.

**Results:**

Results demonstrate that students are legitimately peripherally participating within both medical student CoPs and wider medical CoPs. Themes identified within the narratives have allowed the development of a new model to understand PIF within the context of LPP in a CoP. This has five facets: Awareness, Collaboration, Negotiation, Evaluation and Realisation. Sophisticated reflection-in-action is shown to be an important aspect of PIF and enables a more conscious understanding of the change that is occurring in our students.

**Conclusion:**

PIF is a complex, non-linear process that is supported by reflection-in-action and early student introduction to clinical practice. It can be recognised in students’ narratives in their changing use of language, their understanding of the medical COP, and their evolving relational participation with those around them. This study adds to those that have previously explored PIF. The model of PIF developed in this study illustrates how experiences in the clinical environment support PIF. Medical educators may find this model helpful when considering how PIF can be explicitly encouraged in the medical curriculum and how reflection may be used for the purpose of identity change.

## Introduction

Medical professional identity (PI) is widely recognised as an important goal of medical education [1]. In this paper, medical PI is understood as how a doctor or student thinks of himself or herself as a doctor [2]. It is the feeling of belonging to the medical profession and to a larger body of doctors. It is at times of national crisis that we are reminded of the essential role that medical education has in developing PI. This *belonging* to the profession was recently tested as we called for both new and old members of our medical community to volunteer to take up front-line posts in the NHS [3]. Whilst fostering PI is understood as a worthy attribute of medical education, there remains no clear method of how we actually encourage the development of PI within our students. Much of the existing research focuses on what PI is and why it is important. More recent interest has focused on measures of PI [4–6] Limited studies however explore how PI is formed [7].If medical education is to have an effect, it is important to understand the processes involved in PIF within individuals. This study therefore explores professional identity formation (PIF) in graduate entry medical students in their first year of medical study at a UK based medical school.

Mann recognised [8] that to meet the challenges associated with encouraging PI formation, medical education could not rely on simply improving current contemporary teaching and learning methods. A re-evaluation of medical education was required, one which prioritised participatory learning, that is, learning which is indivisible from its context and embedded in social processes. One way in which this could be achieved is by implementing socio-cultural approaches to learning such as those associated with communities of practice (CoP).

There are various of ways of considering CoPs which are well discussed elsewhere [9, 10]. This study has its foundations in the seminal work of Lave and Wenger and Wenger [11, 12], both of which discuss the trajectory that newcomers take within a CoP through legitimate peripheral participation (LPP). Wenger [12] identified three attributes of a community; (1) mutual engagement, the practice that exists in a community where meaning is negotiated among the participants, (2) joint enterprise, the mutual accountability established through negotiated responses by participants in given community situations and, (3) shared repertoire of resources, artefacts and language development. It is these collective intentions, shared identities and stewarding knowledge domains that distinguishes a CoP from networks, where relationships are linked for example to problem solving and knowledge creation [13]. A CoP gives time and space to understand practice, preventing social life being reduced to such ‘transactions, interactions, and problem-solving activities’ [14].

LPP is a defining characteristic of situated learning, where learning is understood as relational, between people, in a specific social and historical cultural context. It is a ‘conceptual bridge…. inherent in the production of changing persons and changing communities of practice [11]. Knowledgeability, knowledge formed by a person in practice, changes as learners change through relational ongoing practice with ‘diverse others participating differently’ [14]. Through LPP learners change, becoming different people, which is how new identity construction occurs [11].

PI is an internal construct. It is acquired gradually and is subject to constant change. It involves an interplay of individual, collective and relational identity within a CoP [15].It is through such ‘relational participation’ that new perspectives arrive, and legitimate peripheral participants themselves contribute to future changes within their CoP [11]. PI must not be confused with PIF, which is the focus of this study. PIF considers how medical PI develops and is important to understand in order to maximise the development of PI within the medical school environment and beyond. PIF occurs through experiencing practices and filtering the many unique life experiences that individuals have and which continue to shape their identity [16]. The importance of the link between participation, practice, PI and learning in medical education is crucial and is succinctly summarised by Forsyth who comments ‘who they are, influences how they practice’ [17].

PIF in terms of individuals in communities raises issues of agency, that is the capacity of an individual to act as an independent person whilst at the same time participating within a CoP. Lave and Wenger [11] advocate that whole person agency in fact requires learners to engage in many relational roles. This can only be achieved if there is a decentering in relational terms of the individual. Such agency may be achieved by ‘adoption and adaption of different forms of participation and identity construction within different communities of practice’, as discussed by Handley, et al., [18]. This understanding of agency is important when studying LPP as we need to understand how to foster a strong sense of PIF in our students whilst recognising the importance of them also maintain a sense of self.

Within medicine there are multiple specialist communities of practices, such as general practitioners, surgeons and paediatricians [19]. In this way, medicine forms an elaborate landscape of multiple identities and different practices, each with a negotiated social learning history, for example a way of teaching and behaving. There also exists a larger CoP, that of *being a doctor* [20]. This CoP has its own collective identity and it is within this large CoP that these smaller specialty communities exist. Medical students similarly have been shown to form their own CoP [21], whilst also belonging to a wider medical CoP [22]. Recognising the socio-cultural learning, both within and between these communities is necessary to understanding PIF [23].

Socialisation through participation, where meaning is developed through relationships and shared identities [18], and practice, where knowledge and skills take on meaning ‘in accordance with the way of experiencing practice’ [24], is pertinent to PIF. Further research is needed to understand both the nature of PIF and the socialization process by which identity is formed [25].

## Aims

In this study, we sought to gain greater understanding of the experience of being a medical student. Our aim was to use LPP within a CoP to consider PIF.

The following research questions are addressed:

1. What relational interactions occur between individuals during LPP within a CoP?
2. How does LPP help us understand PIF?

## Methodology

### Setting

The current study was conducted at a Medical School that admits life science graduates with at least an upper second-class honours degree to a 4-year MB ChB degree course that runs alongside the traditional 5-year MB ChB programme. Graduate entry (GE) students are taught separately from the 5-year course students in their first year and join them after this point to enter the clinical phase of their medical education. The instruction method during the GE first year is Problem Based Learning (PBL).

The course is organised into 6 themed modules, each lasting three or four weeks. In addition, there is a skills module covering related clinical and communication skills which are delivered throughout the year. Each week, students are given a paper clinical scenario or problem that forms the basis of the weekly study. PBL discussions and learning activities are integrated across subjects and include both biological and social science topics. Students are exposed to clinical medicine from the start of the year and spend one day a week in a general practice clinic. Four students are randomly allocated to each general practice clinic at the start of the year and remain there throughout the year. Teaching and learning opportunities, including patient encounters, are organised according to the teaching theme of the week. Teaching is organised by a practising General Practitioner (GP) who is employed, part time, by the University. The GP is responsible for ensuring consistency of teaching across GP practices. There is a handbook detailing all the activities to be covered by students which include patient contact and presentations. Students also discuss experiences from the clinic in their PBL sessions.

### Participants

At the start of the academic year all new GE students were contacted via e-mail, sent from course administrators, providing an outline of the study. Students were invited to volunteer to participate in the study. It was made clear that this study was not compulsory and was separate from any assessment on the course. Ten GE students gave their written informed consent to participate in the study, from a total cohort intake of 40. These students were then given the opportunity to meet the researchers, the purpose of the research was further explained, and students’ questions were answered. The participants comprised six female and four male GE students. The age range at entry into the study was 22–30 years. Three participants had studied professional degrees such as pharmacy while seven participants had previously studied basic life sciences degrees such as biochemistry. Two participants had postgraduate degrees in addition to their undergraduate degree.

### Instruments for semi-structured group interviews

Semi-structured group interviews were used to gather data. The interview guide was developed to provide consistency of questioning between groups but with the scope to explore responses for more detail where appropriate. The semi-structured interviews primarily concerned three domains:

i. Students’ experience of being in clinical practice.
ii. Students’ experience of their participation with GPs and patients in the clinical setting.
iii. Student engagement with the course and awareness of their PIF.

Interview prompts included questions such as ‘What effect does your clinical experience have on your understanding of medicine?’ and ‘How do you gain an understanding of what being a doctor is about?’. The aim was to encourage consideration of relevant topics whilst allowing scope for the groups’ discussion to develop autonomously.

### Design and procedures’

To keep group numbers small and allow maximum opportunity for participation within the group interviews, students were randomly allocated to one of 2 interview groups (a and b), within which they remained for the duration of the study. Both groups were interviewed at 3 points during their first year of medical student.

The interviews were audio recorded and later transcribed. The first interviews denoted ‘stage 1’, took place after the first module (4 weeks) where all students were new to the course. The second, ‘stage 2’ interviews took place after 17 weeks of study, in February. By this point students had covered four modules, written one set of formative assessments and had changed PBL groups twice. The third, ‘stage 3’, interviews took place in May after 23 weeks of study. At this point students were nearing the end of their first year of studies and were starting to prepare for their end of year, summative exams.

Interviews were conducted by two researchers, both of whom were experienced teachers in higher education, and one of whom who was experienced in social learning.

Analysis.

A thematic analysis of the data was conducted, as described by Braun and Clarke [26]. This involved 6 phases of data analysis.

1. Data from the audio recordings was first listened to by the researchers and later transcribed. Students were allocated a pseudonym to maintain anonymity yet allowing linking of individual student responses at the different interview stages. Researchers then immersed themselves in the data, repeatedly reading and re-reading the transcripts and searching for meanings and patterns in what was being said.
2. Initial codes were generated independently by each researcher and represented sections of text that held meaning either in the semantic or latent sense.
3. These were subsequently discussed between the researchers until agreement was reached as to the overarching themes in the data and the significance of them in relation to the research questions. Thus, the thematic analysis was, ‘theoretical’ in so much as the researchers were searching for evidence of social learning theory in the responses, however the research questions did evolve throughout the coding process allowing for a more inductive analysis.
4. A process of reviewing the themes to ensure that there was both enough data to support them and that they were not overlapping was undertaken. Themes were then defined and named. Particular attention was given to how the themes fitted together and the overall story they told about the data. The term facet was used in presenting the results because individual themes could be considered in more than one way at different interview stages.
5. The themes were examined in the light of existing literature on the topic
6. Ideas were written up.

## Results

**Fig. 1:**
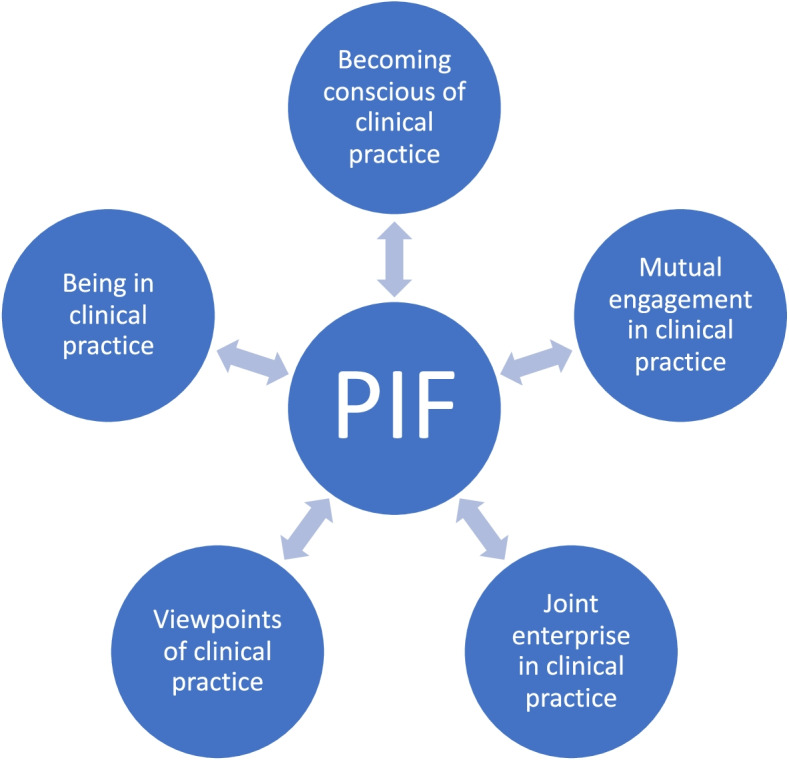
Theorised five-facet model of PIF within a Community of Practice

Our subjects demonstrated that they felt part of a wider medical CoP and specifically that they were legitimately peripherally participating in a CoP with the GPs that they had weekly contact with. The 5 major themes drawn from analysis of the results, hereon referred to as *facets*, allowed the development of a new model to understand LPP and the processes that are involved in the PIF of our cohort of students. This is visually represented in Fig. 1 below. The term facet was used as it captures the complexity of PIF and the notion that its development occurs through engaging with multiple processes in a non-linear fashion.

### Facet 1: Awareness

Many students at Stage 1 interviews understood their learning in clinical practice as an extension of small group teaching. They were not yet appreciating that the clinical environment gave them the opportunity to contextualise their classroom learning and offer a real-life perspective on it. These students were categorised as being in a state of *pre-awareness*.

Some students however conveyed a sense of awareness. They discussed how the clinical environment offered them a different view of medicine. Their narratives describe them being challenged by their experiences and in turn their questioning of existing primary sources of knowledge like lectures and textbooks. This conscious realisation, an awaking, is a necessary aspect of LPP and a key process in PIF. It demonstrates that the lived experience of talking to GPs and being immersed in the clinical environment offers a richness to the learning experiences of our students that is not realised through classroom learning alone. It thus supports the importance that early exposure of medical students to clinical practice has in both contextualising learning and in PIF. This is illustrated in the quote below:

[clinical practice] ‘*is bringing totally different concepts that I’ve not even thought of being linked and new ideas that people haven’t mentioned in lectures and that aren’t necessarily written in the textbooks………when we* [other medical students] *talk to our ladies with breast cancer………..you don’t really understand how it* [illness] *impacts on someone’s life until you see it in front of you…..it makes it so much more real*’. (Student – Elizabeth – Stage 1).

### Facet 2: Collaboration.

Engagement in clinical practice and talking to both GPs and patients in the community appears to enable our students further consider their classroom learning. They are looking to the GPs as knowledgeable others in the CoP [11]. Their narratives suggest coherence through mutual engagement, establishing collective norms, and expectations resulting from a re-examination of importance and meaning. Through this co-operative participation relationships are built, and connections are made that bond members of the community together. Students are, in this sense, legitimately peripherally participating in the GPs CoP and in doing so are developing a sense of how their classroom learning will be used in the real-world. They are aligning themselves with the GPs that they are talking to and in this sense their PIF is occurring.

‘*It’s all very well reading something in a book…when you see patients, you kind of get their priorities…I have a list* [of priorities] *in my head and it just rejigs when I have a conversation with the GP*’ (Student – Rachel—Stage 2 interview).

‘*When a GP says that never happens in GP practice…….that is so off the mark I* [appreciate that and]*……..I’m going to re-evaluate where I put that and prioritise that in my knowledge*’ (Student – Alice – Stage 3 interview).

### Facet 3 – Negotiation

In negotiating a shared understanding of the common purpose of the group, the group’s joint enterprise. Both stage 2 and 3 interviews have students that demonstrate knowledge formed in action, where meaning is negotiated in the context of the CoP and what is important to the CoP is considered. It is this appreciation of reality that appears to be important to them and further aids their legitimacy within the CoP. LPP within the GP’s CoP thus allows students to develop get a sense of who they may be in the future which is an important aspect of their PIF.

[In the clinic you have] *‘the opportunity to try things out…..a phrase or kind of explanation and seeing what response you get back* [from patients]*…it’s like refining your efficiency and also your way of connecting with that person* [the patient]*’ I think the GP practice almost gives us a lengthy amount of time to refine what we’re going to go on to be….potentially experts’.* (Student – Fiona – Stage 2).

Negotiating meaning is key in this process and being part of the CoP provides a safe environment from which to do this. In terms of LPP this creates mutual accountability integral to both Jane and GP.

*‘if you have any…worries or concerns I find our GP’s very good at reassuring us that we’re where we should be at this point and everyone actually feels like that….you can’t ask other people in the same way that you do them’ (Student – Jane – Stage 3 interview)*.

### Facet 4—Evaluation

Through mutual engagement and joint enterprise students develop perceptions on practice. Students may show a mismatch between their peripheral practice and GPs full participation in the community. Reflection is a way of making sense of this and allows students to consider how they may be both part of the CoP whilst maintaining their own sense of agency. Emma comments on her GP’s practice and depicts reflection-in-action [27]. Emma’s viewpoint is evidence of her developing engagement within the CoP and an evolving PIF.

*‘You can look at them [GP] and think well that’s a really good way to do it but you can also think well that’s their way of doing it, but I wouldn’t do it like that. I would maybe do something different. That’s OK to think that.’* (Student—Emma – Stage 2).

Rachel’s trajectory, past and future participation, exemplifies knowledgeability, and a collective becoming. Rachel’s integrated understanding of her world suggests an increasing awareness of PIF and of her LPP within a medical CoP.

[Feeling different] ‘*it is like a really slow transition…just accumulating knowing really slowly… progressing into being a doctor…it’s really a kind of subconscious thing it just kind of filters into you……your vocabulary and demeanour, it does change*’ (Student – Rachel – Stage 3).

### Facet 5 – Realisation

Peripherality is complex to interpret, it is not a physical place within a community [10]. Philip, understands his current knowledge as ‘idealistic’, perhaps acknowledging his current participation as peripheral. His vision of future practice, of other people in full participation who will make his knowledge more ‘realistic’, will occur ‘in the world and in practice’. This exemplifies an identity trajectory generated through a history of practice and a vision of future professional identity [11].

‘I get the impression that our knowledge…. it’s probably still very idealistic and we will be meeting people in A&E who will probably make it much more realistic and bring it much down to what it’s like in the world and in practice’. (Student – Philip – Stage 3 interview). He understands that his participation within the CoP is peripheral and that he needs further experiences to fully contextualise his learning.

Our students are not only learning ‘*from* talk’, they are learning ‘*to* talk’ [10]. This is a key part of a student’s learning trajectory within a CoP and is important to identity development. John now explicitly recognises the complex importance of language and of having a shared repertoire of practice within the medical community, which were not evident in the proceeding interviews. He is thus signalling his conscious participation within the CoP and his developing sense of being a part of that CoP. He is demonstrating his PIF in the use and understanding of language as a shared repertoire within the CoP of medicine.

‘I’m talking to a patient and I’ll actively go ‘oh that’s jargon, I won’t use that. I think it’s important to have two modes [of talking] the more technical mode, [and] a less technical mode’ (Student – John – Stage 3 interview).

Each facet represents what occurs within a student as they legitimately peripherally participate within a community of medical practise and which contributes to PIF. The two-way arrows indicate that PIF is dynamic. Facets are related but in a non-linear fashion. The figure visually demonstrates that facets will need to be visited and revisited as students understanding of the medical world and their place within it develops.

## Discussion

This paper focuses on PIF through relational interactions during LPP in a medical CoP. The longitudinal design of the study allows for the non-linear nature of PIF to be revealed. A new model to further understand the processes involved in PIF within a CoP is presented.

Schematic representation of PIF currently exists [15]. Such representation is helpful as it provides a way of understanding how existing personal identities may be adapted, through socialisation, to allow the development of a PI that is consistent with the responsibilities and expectations of the medical profession. They also help us understand how CoPs and LPP may be used to understand PIF in the medical profession. They do not however provide a depth of meaning as to what occurs in practice, which our longitudinal study offers. LPP has a pivotal role in PIF. It allows participants access to a ‘*nexus of relationships otherwise not perceived as connected’* [11]. Thus LPP, once it is enabled, allows an opening through which participants can explore and learn from full participants within multiple CoPs. PIF only results from participation in practice, and this is the principal reason why LPP differs from passive observational process such as shadowing. With shadowing, students would watch a community *in* practice, they would not *participate in* a community of practice. Students need to participate in practices, such as those indicated in this study, for PIF to occur. Handley [18 p 651], gives helpful guidance in understanding the terms practice and participation, where practice as ‘praxis denotes meaningful engagement in our social communities’. Such engagement in practices as discussed in this study. Participation denotes ‘meaningful activity where meaning is developed through relationships and shared identities. Thus PIF, as shown in this study, occurs through participating in relationships with shared medical identities within a CoP. The interview transcripts in this study indicate that there was constancy of participation in similar practices across all student experiences within different clinics.

This study considers PIF through LPP which contrasts with other approaches to studying PIF. Role models have been discussed as important to PIF [28, 29] and we do not intend here to dispute this notion. Role models however ‘influence and teach by example’ [29]. We suggest that complex processes are involved, particularly where role modelling leads to an unconscious modelling of behaviour. Our model does not therefore exclude role modelling as important to PIF, rather it may shed more light on how role modelling aids PIF.

Our narratives, through the facets of awareness, collaboration, negotiation, evaluation and realisation, indicate that LPP is not characterised by a unidirectional and stepwise progression in relational participation in practice. The facets of collaboration, negotiation and evaluation were visited and re-visited by students in stage 2 and 3 interviews, indicating the motion away from the periphery as part of the legitimate participation. Collaboration provides an example of a facet that is described by students in both stage 2 and 3 interviews. Both Rachael and Alice indicate how they are reflecting on their interactions at the GP practice and using these experiences to reframe their understanding. Alice’s conversation with the GP however indicates a more complex participation compared to Rachel’s. Each student requires the GP to participate differently with them. This suggests that, while facets may be re-visited, the quality of that experience is different each time and reflects a developing PIF.

Interestingly, Facet 1, awareness, and Facet 5, realisation, had only Stage 1 or Stage 3 interviewees respectively. This may indicate that students need a certain amount of conscious understanding of the experiences that they are engaging in before they can participate within a CoP. It appears that developing this awareness enables LPP that, prior to this, may not have been fully operational. It is the inaugural step into becoming a legitimate member of a CoP and thus allows the process of PIF to begin. Conversely, ‘realisation’ may only be possible after visiting the other facets. We do not suggest here that Facet 5 reflects full participation within a CoP, as clearly this would not be possible of a student. Rather we suggest that these facets provide a theoretical framework for understanding the processes that occur during LPP within a CoP and offer a way to better understand PIF in medical students. The facets must be visited and revisited multiple times over the course of a medical school career and beyond. Each time the depth of the experience is much greater and carries with it a different meaning or interpretation than it did before. Thus, over time, a medical student moves closer to full participation within the CoP. This movement to full participation would not be possible if a student were passively ‘shadowing’, and this heightens the emphasis of participation.

PIF is recognised in the narratives in several ways. The students’ use of language alters over time and indicates that they are legitimately peripherally participating within medical CoPs. Their narratives demonstrate that they are starting to feel like doctors. The students’ emerging understanding of the medical profession, its’ historical context and the students’ future role within, it is also evident. Both factors give an explicit indication that PIF is occurring. Through the students’ reflections we can see the evolving relational interactions between the student, the patient, and the GP and this is further observable evidence of the process of PIF. Emma’s Facet 4 quote illustrates reflection-in-action [27]. It demonstrates how Emma is making a conscious judgement about how she will do things in the future. From a PIF perspective this is important. She is taking ownership over her actions and considering ways of making the practice her own. In doing so, she is challenging the old knowledgeabilities, demonstrating the evolving nature of a CoP [30]. Thus, individuals are not only shaped by the CoPs that they participate within but themselves change the CoP. It is this participation in practice which is both active and evolving, that appears to be key to PIF. The students do not watch what others in the community are doing, they become part of the community. They engage in discussions with peers, GPs and patients, reflect upon this discussion and in doing so consider their meaning and the relevance to their own practice. This allows the process of PIF to evolve and brings new meaning to the students’ peripheral place within the community. Our students are experiencing LPP in the lived-in world in relational ongoing social practice, where changing knowledgeable skill becomes part of the changing identity process [11].

An argument that has been made against the participatory learning within a CoP concerns whether or not situated knowledge can be transferred to new learning settings [31]. We illustrate how our facets of LPP are not isolated from one another, and that relationships experienced in one are not unconnected from relationships experienced in another. Lave [32] refers to complex interconnectedness of social systems and Lave and Wenger [11] refer to LPP having an interconnectedness. Our students’ PIF appears to occur through such interconnectedness. It therefore seems likely that, as students move from one medical CoP to another and from one clinical attachment to another, their reflection on experiences in other CoPs will enable a richer engagement experience of LPP within a new CoP. PIF is an ongoing process. It does not end when a student graduates as a doctor or when they become a full participant in the CoP. PIF will continue to evolve throughout an individual’s lifetime in a dynamic fashion and it is possible that our facets are visited even by ‘old-timers’ [11] in a CoP when a new situation is encountered that challenges a previously held belief about the CoP that they are operating within.

With a dramatic recent shift to virtual learning environments and rapid change necessitated by the recent pandemic situation, it is important that PIF is not forgotten. Medical educators need to recognise the role that LPP within multiple CoPs has on PIF. The likely reduction in early exposure to the clinical environment as a result of the current pandemic must therefore be seen not only as reducing the ability for students to gain the practical skills associated with becoming a doctor. Medical educators also need to recognise the reduced opportunities in terms of LPP within medical CoP and therefore opportunity for PIF. Medical educators can also do more to promote PIF in their medical student cohorts. PIF is not a passive process. It does not happen *to* a student. The student themselves is the agent of change. PIF is thus a pursuit over which the student themselves can take ownership and medical educators could consider endorsing this notion. Reflection is also key. This study gives further insight into its central importance to the medical profession. Reflective practice not only improves the quality of patient care [33], it also helps a medical student to *become* a doctor. Educators can help students by promoting reflection for the purpose of identity change.

## Limitations of study

This is primarily a small sample study, necessarily unique in order to allow adequate focus of the individual narratives of the students as well as an understanding of how they fitted together. It is acknowledged that the sample was sourced exclusively from a graduate entry cohort of self-selecting students so they may have had an interest in reflecting on how they learn. Students also all attended a single institution, so generalizability of results is not claimed. Nevertheless, the results offer some insight into the nature of the learning and PIF that may be occurring during the early stages of LPP within an established community of practice. Explicit motivation to participate was felt to be important in order to explore the narratives on this topic. Researchers were faculty members and known to the students which had the potential to influence what was said, however participants were explicitly advised that their participation in the study was voluntary and entirely separate to their participation in the course. Moreover, it was felt that the researchers in depth knowledge of the course allowed for good contextualisation within the research. An independent researcher, who was knowledgeable of the PBL process but not involved in the original collection of data was involved in the analysis of the results and in the write up of the paper.

## Conclusion

Medical PIF is a complex process. This study uses LPP to better understand the relational nature of PIF in graduate entry medical students over their first year of medical study. Social interaction and participation within medical CoPs afford opportunities for new experiences that in turn brings about change in the students’ themselves. This change, which is understood as PIF, can be recognised in the students’ narratives both in the language that they use and the experiences that they describe. A model proposing the process involved in PIF is presented. We propose that this model can be used to better understand the trajectory of PIF in medical students as they engage with the clinical practice of medicine. We demonstrate that PIF in a non-linear process. Facets are visited and re-visited over time, but the quality and depth of experience is different as the process of PIF occurs. We thus postulate that the identity change is not situated within an isolated environment or a single CoP but can be translated and further developed as the participant moves between CoPs. Reflection is also shown to be important to the process of PIF. It not only enables PIF to occur but may also help the student to consciously recognise the active role that they have in their own PIF.

## Data Availability

The datasets used and/or analysed during the current study available from the corresponding author on reasonable request.
